# Melatonin Action on the Activity of Phagocytes from the Colostrum of Obese Women

**DOI:** 10.3390/medicina55100625

**Published:** 2019-09-23

**Authors:** Tassiane C. Morais, Adenilda C. Honorio-França, Mahmi Fujimori, Ocilma B. de Quental, Rafael S. Pessoa, Eduardo L. França, Luiz C. de Abreu

**Affiliations:** 1 Postgraduate Program in Public Health, Faculdade de Saúde Pública, Universidade de São Paulo (USP), São Paulo 01246-904, Brazil; tassi.morais@usp.br (T.C.M.); luizcarlos@usp.br (L.C.d.A.); 2 Laboratório de Delineamento de Estudos e Escrita Científica, Centro Universitário Saúde ABC, Santo André 09060-870, Brazil; ocilmaquental2011@hotmail.com; 3 Institute of Biological and Health Science, Universidade Federal de Mato Grosso (UFMT), Barra do Garças, Mato Grosso 78600-000, Brazil; adenildachf@gmail.com (A.C.H.-F.); mahmi_fujimori@yahoo.com.br (M.F.); faelpessoa@gmail.com (R.S.P.); 4 Department of Nursing, Faculdade Santa Maria (FSM), Cajazeiras 58900-000, Brazil; 5 Programa de Mestrado em Políticas Públicas e Desenvolvimento Local da Escola Superior de Ciências da Santa Casa de Misericórdia, Vitória 29045-402, Brazil; 6 Graduate Entry Medical School, University of Limerick, V94 T9PX Limerick, Ireland

**Keywords:** body mass index, breastmilk, colostrum, phagocytes, melatonin, obesity, oxidative stress

## Abstract

*Background and objectives:* Breastfeeding promotion is an important public health strategy for counter-balancing the negative effects of maternal overweight and obesity. Colostrum contains melatonin, which can attenuate the impacts of excessive maternal weight and boost the infant’s immune system. Therefore, the objective of this study was to analyze the effects of melatonin on mononuclear (MN) phagocytes from the colostrum of women with pre-gestational obesity. *Materials and Methods:* Colostrum samples were collected postpartum from 100 women at a public hospital in São Paulo, Brazil. The donors were divided into two groups: the control group and the high body mass index (BMI) group. Melatonin levels in the colostrum were determined by an ELISA Kit, and the functional activity of MN cells was assessed using the phagocytosis assay by flow cytometry, and reactive oxygen species (ROS), intracellular calcium, and apoptosis were assessed by fluorimetry using a microplate reader. *Results:* The colostrum of mothers with pre-gestational high BMI exhibited higher melatonin levels (*p* < 0.05) and lower phagocytosis (*p* < 0.05) and ROS release (*p* < 0.05). Superoxide release was similar between the normal and high BMI groups (*p* > 0.05). Intracellular calcium release and apoptosis were also higher in the high BMI group (*p* < 0.05). Melatonin levels likely increased the phagocytosis rate and reduced intracellular calcium release and the apoptosis index (*p* < 0.05). *Conclusions:* The results suggest that melatonin is a possible mechanism for maternal–infant protection against obesity and restores the functional activity of colostrum phagocytes in obese mothers.

## 1. Introduction

Obesity is a complex public health problem that can emerge in childhood [1]. The World Health Organization estimates that 2.8 million people die every year from health conditions caused by overweight. Actions directed toward preventing excess weight gain could reduce this number [2]. 

Light exposure at night is an important factor contributing to the development of obesity and dyslipidemia because it reduces the endogenous production of melatonin [3]. Melatonin, an indolamine produced mainly by the pineal gland, plays an important role in the temporal regulation of biological rhythms [4,5]. Melatonin, one of the hormones contained in milk, plays an important role for infants [6,7,8,9]. Studies suggest that a decline in melatonin levels is associated with dyslipidemia and obesity. Thus, melatonin is a potential candidate for controlling excess weight gain and associated metabolic diseases [3,10,11,12].

The anti-obesogenic and the weight-reducing effects of melatonin [4] appear to be associated with the control of circadian rhythms in peripheral adipose tissues [12]. Melatonin can modulate immune responses, thereby highlighting the interaction between the immunological system and metabolic pathways [13]. The beneficial effects of this interaction are particularly important in enhancing the immune response of obese individuals because they are more susceptible to infections. The mechanisms underlying this response are not fully understood, but earlier studies suggest an association between adipose tissue metabolism and the activity of immune system components [14,15,16].

Obesity has increased in women of reproductive age, and overweight during pregnancy has been associated with several maternal and fetal complications [17]. Changes resulting from maternal overweight also appear to be reflected in the biochemical, hormonal, immunological, and nutritional components of human colostrum and milk [18,19,20,21]. Colostrum is rich in immunologically active cells, and the changes that obesity cause to its composition may affect the functional activity of the phagocytes contained therein.

Colostrum phagocytes are important protective factors for infants [8,22,23], and in neonates, they are an important protection against gastrointestinal and respiratory infections [23,24]. Although they can be modulated by bioactive components such as hormones, the effects of melatonin on the colostrum phagocytes of obese mothers remain unknown. As such, the aim of this study was to analyze the interactive effects of melatonin on the functional activity of the colostrum phagocytes of high body mass index (BMI) women.

## 2. Materials and Methods

### 2.1. Subject

Maternal colostrum from 100 donors was evaluated in a cross-sectional study of the maternity ward at the Hospital of the University of São Paulo, SP, Brazil, in 2017. The inclusion criteria of the study were as follows: pre-gestational weight known or measured until the end of the 13th gestational week; gestational age at delivery between 37 and 41^6/7^ weeks; negative serological reactions for hepatitis, HIV, and syphilis; to have performed prenatal health care; to have to maternal dietary restriction during pregnancy and postpartum period; and to have signed the informed consent form. The exclusion criteria were as follows: gestational diabetes; twin pregnancy; and fetal malformations.

Participants were divided into two groups according to their pre-gestational BMI (calculated as weight (kg) divided by the square of the height (m^2^): the normal BMI group (*n* = 50 and a BMI between 18.5 and 24.9 kg/m^2^) subjects were recruited as the control; and the high BMI group (*n* = 50 and BMI ≥ 30.0 kg/m^2^).

This study was approved by the Institutional Committee for Ethics in Research of the Hospital of the University of São Paulo (HU/USP) (CAAE 46643515.0.3001.0076), and all the subjects gave informed written consent before entering the experimental protocol.

### 2.2. Colostrum Sampling and the Separation of Colostral Cells

About 5 mL of colostrum from each woman was collected manually into sterile plastic tubes between 48 and 72 h postpartum. Colostrum was collected during the daytime (between 10:00 a.m and 12:00 a.m.). The samples were centrifuged (160× *g*, 4 °C) for 10 min, which separated the colostrum into three different phases: the cell pellet, an intermediate aqueous phase, and a lipid-containing supernatant. The upper fat layer was discarded, and the aqueous supernatant was stored at −80 °C. Cells were separated by the Ficoll–Paque gradient (Pharmacia, Upsala, Sweden), producing preparations with 98% pure mononuclear cells, analyzed by light microscopy. Purified macrophages were resuspended independently in a serum-free medium 199 (Gibco, Grand Island, NE, USA) at a final concentration of 1 × 10^6^ cells/mL. The cells were used for the assays, and the colostrum supernatant was stored at −80 °C for later hormonal analysis.

### 2.3. Determination of Hormone

Melatonin was extracted from the aqueous supernatant by affinity chromatography, concentrated in speed-vacuum and determined by a commercial ELISA kit (IBL, Hamburg, Germany). The reaction rate was measured by absorbance in a spectrophotometer equipped with a plate-reader and a 405 nm filter. The melatonin levels were calculated as a function of the standard curve in pg/mL [7].

### 2.4. Treatment of Colostral Phagocytes with Melatonin

To assess the effect of melatonin on the phagocytes, reactive oxygen species, viability, and intracellular Ca^2+^ release, mononuclear (MN)phagocytes were induced into an inflammatory process via bioparticles of Zymosan (from *Saccharomyces cerevisiae*). The MN cells and Zymosan were incubated for 2 h at 37 °C and treated with 199 medium (Gibco, Grand Island, NE, USA; negative control) and melatonin (Sigma, St Louis, MO, USA; final concentration 100 ng/mL). The phagocytosis assays were performed with Zymosan pHrodo Green^®^ (Thermo Fisher, Carlsbad, CA, USA). Free radical release, intracellular calcium, and apoptosis assays were performed with Zymosan (Sigma, St Louis, MO, USA) without conjugated fluorochrome to avoid interference in the fluorescence intensity with the other reagents.

### 2.5. Phagocytosis ASSAY

Colostrum MN phagocytes were incubated with pHrodo Green^®^ zymosan particles (Thermo Fisher, Carlsbad, CA, USA) according to the manufacturer’s guidelines. The pHrodo Green® dye was used to determine the phagocytosis rate because it fluoresces bright green under the acidic pH produced during phagocytosis. The 10,000 MN phagocytes were examined by flow cytometry using FACS Calibur (BD Biosciences, San Jose, CA, USA), with an excitation/emission of 509/533 nm. The results were expressed by the Phagocytosis Index (%). The experiments were performed in duplicate.

### 2.6. Analysis of Reactive Oxygen Species

Dihydrorhodamine 123 (DHR 123), which fluoresces in the cell after being oxidized by reactive oxygen species, was used to detect OH and H_2_O_2_. The MN phagocytes were incubated with DHR123 (5 µ/mL; Sigma, St Louis, MO, USA), following the protocol standardized by Radogna et al. [25]. Fluorescence intensity was measured on a Fluoroskan Ascent FL^®^Microplate reader (Thermo Scientific, Vantaa, Finland), with a 485 nm excitation filter and emission filter of 538 nm. The intensity of the fluorescence emitted is proportional to the production of the reactive oxygen species. The results were expressed as the DHR123 mean fluorescence intensity.

The superoxide release was determined by cytochrome C (Sigma, St. Louis, MO, USA) reduction. After the MN cell treatment, the cells were centrifugated and then resuspended in PBS containing 2.6 mM CaCl_2_, 2 mM MgCl_2_, and cytochrome C (Sigma, St Louis, MO, USA; 2 mg/mL). The suspensions (100 μL) were incubated for 60 min at 37 °C on culture plates. The reaction rates were measured by absorbance at 550 nm, and the results were expressed as nmol/O_2_^−^. All the experiments were performed in duplicate.

### 2.7. Apoptosis Assays

The apoptosis index was analyzed using FITC Annexin V (BD Biosciences, Erembodegem, Belgium). Fluorescence intensity was measured on Fluoroskan Ascent FL^®^Microplate reader (Thermo Scientific, Vantaa, Finland), with the 485 nm excitation and 538 nm emission filters. The results were expressed as a percentage of apoptosis. All the experiments were performed in duplicate.

### 2.8. Intracellular Ca^2+^Release Determined by Fluorescence

Cells treated with zymosan and medium 199(Gibco, Grand Island, NE, USA) or melatonin (Sigma, St Louis, MO, USA) were incubatedin the presence of a 5µL Fluo-3 AM solution (Sigma, St Louis, MO, USA). The cells were washed (160 × g, 10 min, 4 °C) and resuspended in a HBSS (Hank’s Balanced Salt Solution) containing bovine serum albumin (BSA) and analyzed by Fluoroskan Ascent FL^®^Microplate reader (Thermo Scientific, Vantaa, Finland), with the 485 nm excitation and 538 nm emission filters. The results were expressed as the mean fluorescence intensity of Fluo-3 AM. The experiments were performed in duplicate.

### 2.9. Statistical Analysis

The data are expressed as the mean ± standard deviation (SD). Statistical analyses were performed with the BioEstat^®^ version 5.0 software (Mamirauá Institute, Belém, Brazil). A D’Agostino normality test and variance analysis (ANOVA) were used, followed by Tukey’s test. A *p* value of <0.05 was considered statistically significant.

## 3. Results

Clinical data for the mothers and newborns are shown in Table 1.

**Figure 1:**
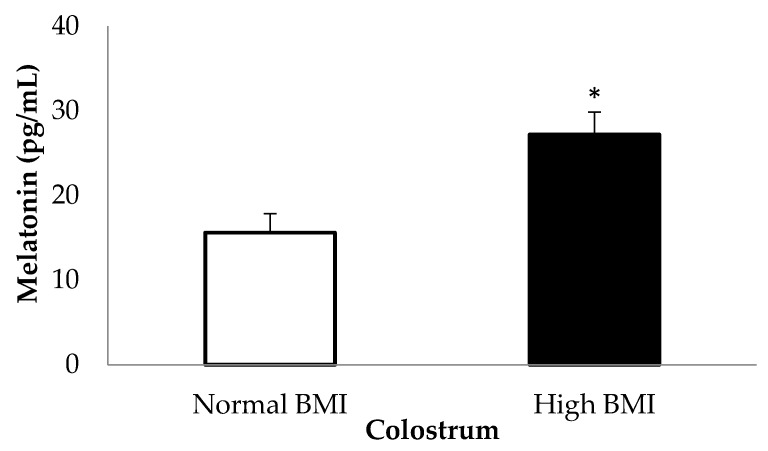
Melatonin (pg/mL) levels in the colostrum of mothers with normal or high BMI pregestational. * Assessed by ANOVA and Tukey’s test. * *p* < 0.05 Statistical difference between normal BMI and high BMI group. BMI, Body Mass Index; ANOVA, A D’Agostino normality test and variance analysis.

Colostrum from the high BMI group had higher melatonin (Figure 1).

**Figure 2:**
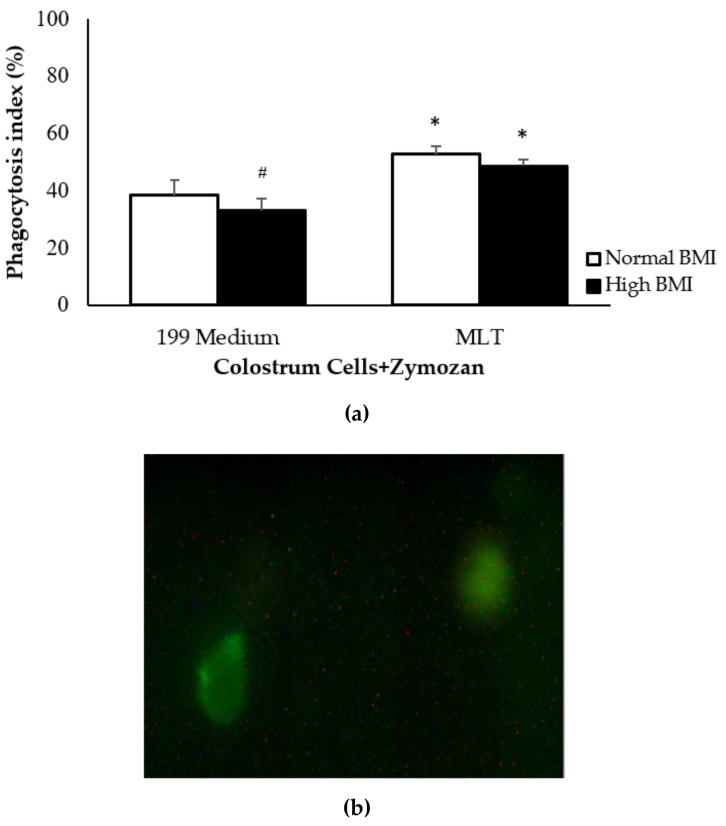
High BMI effects on colostrum mononuclear (MN) phagocytes. (**a**) Phagocytosis rate (%) in colostrum MN cells, from normal and obese women, incubated or not with melatonin. (**b**) Fluorescence microscopy image of pH Rodo^®^ Green zymosan phagocytosis by MN cells, after 2 h of incubation (scale: 5 μm, 100 x—panel). The results are expressed as the mean ± standard deviation (SD). * statistical difference (*p* < 0.05) between control cells (199 medium) and cells incubated with zymosan (Zy) or zymosan and melatonin (Zy + MLT) within a same group. #Statistical difference among groups with the same treatment and sample (*p* < 0.05).

Zymosan phagocytosis by MN cells was lower in the high BMI group than in the control group, and higher after treatment with melatonin, regardless of maternal BMI (Figure 2).

**Figure 3:**
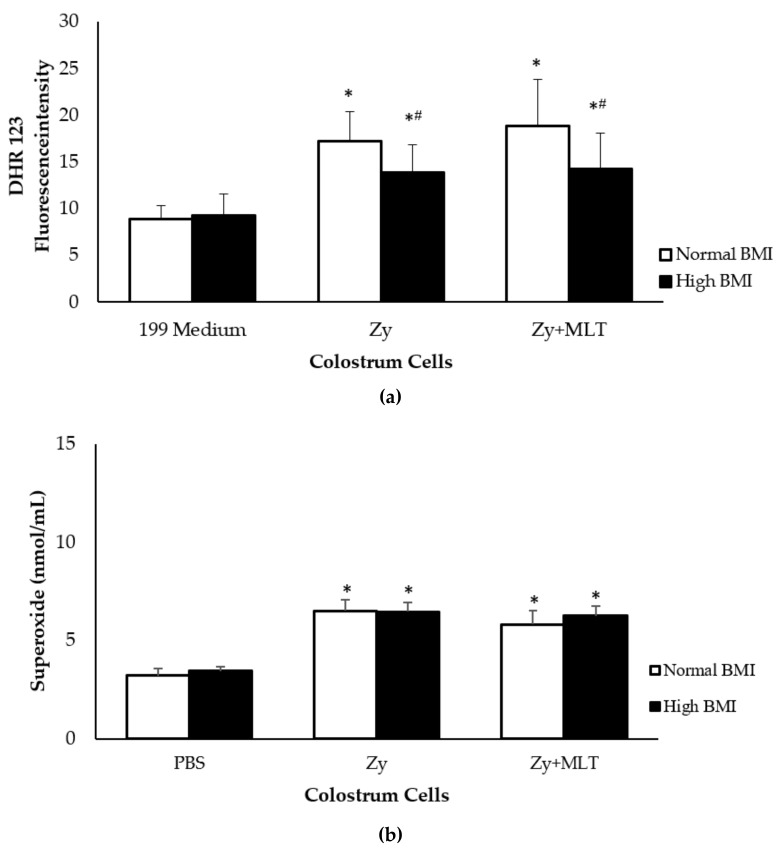
Reactive oxygen species release by colostrum phagocytes. (**a**) Fluorescence intensity of DHR123. (**b**) Superoxide release (nmol/mL). Colostrum phagocytes were incubated with medium 199 or Phosphate Buffer Solution (PBS), melatonin (100 ng/mL) and/or zymosan. DRH123 was analyzed using Fluoroskan Ascent FL^®^ Microplate. The results are expressed as the mean ± standard deviation (SD). * Statistical difference (*p* < 0.05) between control cells (PBS or 199 medium) and cells incubated with zymosan (Zy) or zymosan and melatonin (Zy + MLT), within the same group; #Statistical difference among groups with the same treatment and sample (*p* < 0.05).

The spontaneous release of the reactive oxygen species (ROS) was similar among the groups (*p* > 0.05). In the DHR123 assay, the MN phagocytes of the high BMI group released a lower concentration of free radicals in the presence of zymosan, regardless of the treatment with the hormone. Spontaneous superoxide release by colostrum phagocytes was similar among the groups, and regardless of treatment, this release increased in the presence of zymosan (Figure 3).

**Figure 4:**
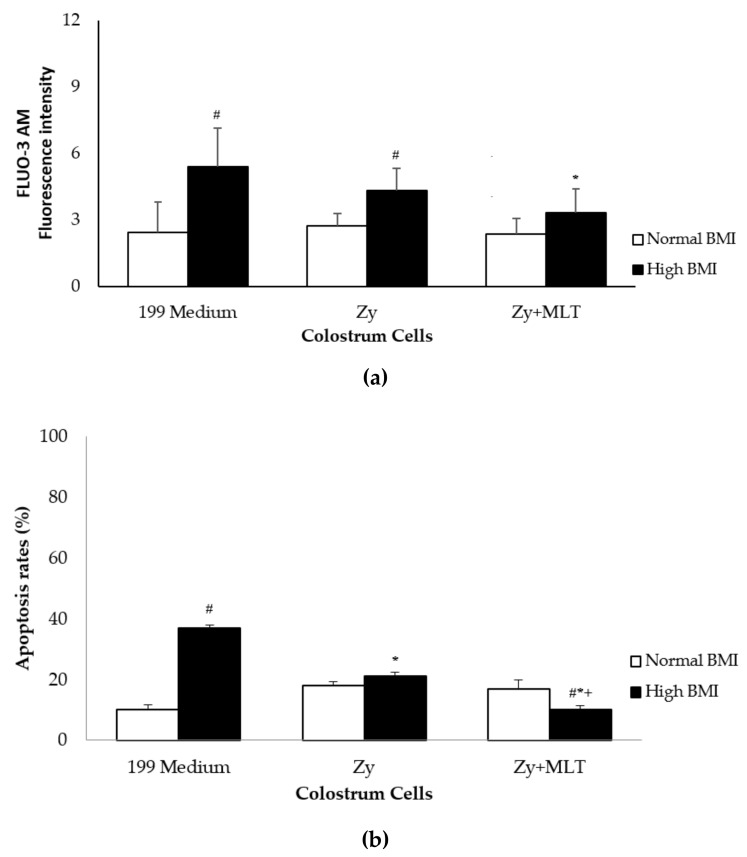
Intracellular calcium release (**a**) and apoptosis rates (**b**) by colostral phagocytes. The phagocytes were incubated with 199 medium, Zymosan, and melatonin (100 ng/mL). The intracellular calcium release was performed with Fluo-3AM (*n* = 10). The apoptosis assay was performed with Annexin V-FITC staining. The results are presented as the mean ± standard deviation (SD). * *p* < 0.05, * statistical difference (*p* < 0.05) between the control cells (199 medium) and cells incubated with zymosan (Zy) or zymosan and melatonin (Zy + MLT) within the same group. +Statistical difference (*p* < 0.05) between cells incubated with zymosan, treated or not with melatonin, within the same group. # The statistical difference among groups with the same treatment and the sample.

Intracellular calcium release was higher in colostrum MN phagocytes from the high BMI group, incubated or not with zymosan, but it decreased when the phagocytes were incubated with melatonin (Figure 4). Colostrum MN phagocytes from high BMI mothers exhibited a high apoptosis index, but this rate declined when they were incubated with zymosan, regardless of their treatment with the melatonin. The lowest apoptosis index was found in colostrum phagocytes from high BMI mothers, incubated with both zymosan and melatonin (Figure 4b).

## 4. Discussion

The impact of maternal obesity does not end with the birth of the child. Maternal obesity combined with hormonal activity and metabolic diseases may affect the bioactive components of breast milk [26]. In the present study, colostrum from women with pre-gestational obesity showed higher melatonin levels, and this hormone affected the functional activity of colostrum phagocytes.

Obese individuals have a significant reduction in melatonin levels [6] in addition to containing melatonin receptors (MT2) [27] in their adipose tissue cells capable of inhibiting adipogenesis [28]. In the present study, the high melatonin levels in colostrum produced by obese women suggest that this hormone participates in an alternative mechanism to control infant obesity.

Another important role of melatonin is to modulate the phagocytic activity of macrophages [7,29]. Studies associating phagocyte activity with excess weight are controversial. Some studies report that obesity decreases phagocyte activity [29] while others suggest that it increases phagocytosis in macrophages and granulocytes [30].

In the present study, bioparticle phagocytosis decreased in the high BMI group. When colostrum phagocytes were treated with melatonin, however, the phagocytosis rate was similar to that of the control group, suggesting that melatonin can restore the phagocytic activity of colostrum cells.

Melatonin seems to promote the phagocytic activity of colostrum phagocytes, which is essential to protecting a newborn in its first days of life [7,8,29]. Melatonin acts mainly via MT1 and MT2, which are both G protein-coupled receptors. In colostrum MN phagocytes, melatonin acts directly via the MT2 receptor, improving efficiency by increasing the expression of dectin-1, a key protein in the innate immune response [29].

One of the main processes in phagocytosis is the formation of reactive oxygen species (ROS), which account for the microbicide activity of phagocytes [31]. Other studies report that obesity is associated with a rise in the oxidative burst of macrophages and neutrophils [32]. However, overweight may be not associated with an increase in oxidative stress markers such as the superoxide anion [33]. Interestingly, in this study, colostrum cells from the high BMI group had a lower ROS release in the presence of zymosan, regardless of the treatment with melatonin, but increased ROS release when incubated only with the hormone. Considering the levels of superoxide anion released in both the control and the high BMI groups, and the phagocytosis-stimulating hormone, the results suggest that, despite their reduction in ROS production, phagocytes can effectively contribute with protective mechanisms of action against infections.

In colostrum MN phagocytes, melatonin may increase the superoxide release during bacterial phagocytosis. However, melatonin can have an antioxidant effect on the presence of metabolic disorders, such as diabetes [7,8]. The distinct effects of melatonin on reactive oxygen species, (cytoprotective or cytotoxic effects) in biological systems depend on numerous factors, such as the dose, the cellular targets, and the time of exposure [34].

One point to be highlighted is that, despite the lower functional activity of colostrum phagocytes from obese women, melatonin acts on the activation of these cells. They increase ROS levels, superoxide release, and phagocytic activity, which seems to compensate for the possible impacts caused by excess weight on the protective mechanisms of breastfeeding. Melatonin, however, may trigger cascades of intracellular events involving the inhibition of cyclic AMP production and intracellular calcium release [35].

In the present study, intracellular calcium release increased in colostrum cells from obese women. The treatments with melatonin reduced intracellular calcium release, suggesting that these hormones act as antioxidants, avoiding cell damage.

An increased release of intracellular calcium is associated with apoptosis induction [36]. Melatonin modulates leukocyte apoptosis via stimulation of MT1/MT2 receptors and relocation of the anti-apoptotic protein Bcl-2 to the mitochondria [25]. Colostrum cells from the high BMI group exhibited a high apoptosis rate, but this rate declined in the presence of zymosan, regardless of its treatment with the hormone. These findings may be associated with increased levels of saturated fatty acids, which can activate inflammatory pathways, mainly by raising caspase activity [37]. As a result, the basal levels of intracellular calcium may increase, as observed in the monocytes of overweight individuals [38].

Although a higher basal level of apoptosis and intracellular calcium was found in the high BMI group, these levels did not impair the immune response via phagocytosis. In the colostrum, more than 90% of the MN phagocytes are formed by viable cells [39], which have an apoptosis rate of nearly 6.7% [36]. Therefore, a significant number of viable cells is available to support the development of an immune response.

Another important factor to underscore is that although zymosan induces an inflammatory process, it may also regulate macrophages via the dectin-1 receptor, thereby producing a regulatory-like phenotype [40]. In addition, interactions with melatonin may favor a non-inflammatory environment, given the antioxidant and anti-inflammatory action of this hormone [41]. Indeed, this association minimizes the possible impacts of maternal obesity on the function of human colostrum phagocytes by modulating the immune response.

Interestingly, in the colostrum, the melatonin peak is at 24 h [7]. The rhythmicity of the secretion of melatonin may be have a significant influence on the functional activity of MN colostrum cells, since the hormone is able to modulate the activity of these cells. This mechanism is likely important for the development of the child, since a significant number of infectious diseases are linked to the type of perinatal nutrition. Thus, the prevention of possible infections may be associated with natural components of breast milk.

Despite the differences in the immunological constituents of the colostrum caused by maternal obesity, breastfeeding should be encouraged, mainly because in vivo colostrum phagocytes will be submerged in a supernatant with different melatonin concentrations. This study is the first to describe melatonin concentrations in the colostrum in women with pre-gestational obesity. However, its cross-sectional design produces limitations. New perspectives could be developed to illustrate the impact of obesity on colostrum melatonin levels over a 24 h period and the impact of obesity and overweight on child health during the first year of life. Thus, we could use free demand breastfeeding itself as a key for intervention strategies. Indeed, breastfeeding continues to be an important public health strategy to combat the obesity epidemic, with promising potential to provide protective effects against obesity for both mother and child [2].

## 5. Conclusions

Colostrum from high BMI groups showed higher melatonin concentrations, and MN phagocytes showed a lower phagocytosis rate and ROS release, but normal superoxide release. These groups also exhibited an increase in intracellular calcium release, which may account for the higher apoptosis index. However, the treatments with melatonin restored the phagocytosis rate and reduced intracellular calcium release and the apoptosis index. These results indicate that melatonin may prevent cell damage in the colostrum cells of high BMI women via its antioxidant action, and this may represent a mechanism of maternal protection against childhood obesity.

## Figures and Tables

**Table 1:** Maternal and neonatal characteristics according to pre-pregnancy body mass index (BMI) group (normal BMI or high BMI).

Maternal and Child Characteristics	Normal BMI	High BMI
Age (years)	26.7 ±5.5	26.6 ±5.7
Diabetes or gestational diabetes (%)	0 0.0%	0 0.0%
Pregestational maternal BMI (kg/m^2^)	21.7 ±1.9	31.3 ±3.9 *
Delivery BMI (kg/m^2^)	26.3 ±2.9	35.2 ±3.7 *
Gestational weight gain(kg)	11.3 ±3.9	9.3 ±3.6
Gestational age at delivery (weeks)	38.9 ±1.1	38.8 ±1.0
Infant sex—male (%)	26 52.0%	28 56.0%
Birth weight (g)	3324 ±428	3346 ±495
Birth height (cm)	46.7 ±3.5	47.4 ±3.3

Maternal and neonatal data are shown as the mean ± standard deviation (SD) or the number and percentages (%) from 50 mothers from each group. They were assessed by ANOVA and Tukey’s test. * *p* < 0.05 = a statistical difference between the normal BMI and high BMI groups. BMI, Body Mass Index; ANOVA, A D’Agostino normality test and variance analysis.
